# In vitro plantlet regeneration from nodal segments and shoot tips of *Capsicum chinense* Jacq. cv. Naga King Chili

**DOI:** 10.1007/s13205-011-0025-5

**Published:** 2011-09-27

**Authors:** Mechuselie Kehie, Suman Kumaria, Pramod Tandon

**Affiliations:** Plant Biotechnology Laboratory, Department of Botany, Centre for Advanced Studies, North-Eastern Hill University, Shillong, 793022 India

**Keywords:** *Capsicum chinense* Jacq., Nodal segments, Plant regeneration, Shoot tips

## Abstract

An in vitro regeneration protocol was developed for *Capsicum chinense* Jacq. cv. Naga King Chili, a very pungent chili cultivar and an important horticultural crop of Nagaland (Northeast India). Maximum number of shoot (13 ± 0.70) was induced with bud-forming capacity (BFC) index of 10.8, by culturing nodal segments in Murashige and Skoog (MS) medium supplemented with 18.16 μM Thidiazuron (TDZ) followed by 35.52 μM 6-benzylaminopurine (BAP). Using shoot tips as explants, multiple shoot (10 ± 0.37) (BFC 8.3) was also induced in MS medium fortified with either 18.16 μM TDZ or 35.52 μM BAP. Elongated shoots were best rooted in MS medium containing 5.70 μM indole-3-acetic acid (IAA). Rooted plantlets thus developed were hardened in 2–3 weeks time in plastic cups containing potting mixture of a 1:1 mix of soil and cow dung manure and then subsequently transferred to earthen pots. The regenerated plants did not show any variation in the morphology and growth as compared to the parent plant.

## Introduction

C*apsicum chinense* Jacq. cv. Naga King Chili is an important spice crop of India belonging to the family Solanaceae. It is a self-pollinated species; however, the occurrence of high cross pollination leads to the formation of variants within Naga King Chili. *C. chinense* is a very pungent chili, measuring 1,001304 Scoville Heat Units (SHU). It is locally called as Naga King Chili (*Bhoot jolokia* or *Naga jolokia* in Assamese), and is native to North-Eastern India more particularly to Nagaland (Bhagowati and Changkija 2009).

In vitro plant regeneration from cells, tissues and organ cultures is a prerequisite for the application of plant biotechnology to plant propagation, plant breeding and genetic improvement. It is the only technology for the production of large quantities of “elite” planting material so as to increase the production and productivity. Micropropagation is advantageous over traditional propagation as it can be used to multiply novel plants, such as those that have been genetically modified or bred through conventional plant breeding methods. It is also used to provide a sufficient number of plantlets for planting from a stock plant which does not produce seeds or respond well to vegetative reproduction. It also leads to simultaneous accomplishment of rapid large-scale propagation of new genotypes (Tandon and Kumaria 2005). The conventional method of chili plant propagation using seeds is restricted by the short span of viability and low germination rate of seeds. Chili plants are also highly susceptible to fungal and viral pathogens (Morrison et al. 1986). Since chili plant lacks natural vegetative propagation, plant tissue culture technique provides an alternative method of propagating novel genotypes. The establishment of efficient and promising protocol for in vitro plantlet regeneration of *Capsicum* is required for the application of modern biotechnological tools, such as asexual reproduction of elite stocks, recovery of useful somaclonal variants, germplasm preservation as well as the production of transgenic plants with improved agronomic traits, interspecific hybrids, and haploid plants (Ezura 1997; Aguado-Santacruz et al. 2004). Though several attempts have been made on in vitro regeneration in the genus *Capsicum,* most of the reports are attempted on *Capsicum annum* (Agrawal et al. 1989; Ramirez-Malagon and Ochoa-Alejo 1996; Venkataiah et al*.*^2003^; Khan et al. 2006) and a few on *Capsicum frutescens* (Subhash and Christopher 1988; Reddy et al. 2002). Only one report exists on in vitro propagation of *C. chinense*, wherein only six shoots per explant were obtained (Sanatombi and Sharma 2008). In this study, we present protocol for in vitro regeneration of *C. chinense* using both shoot tips and nodal segments resulting in higher number of shoots per cultured explants.

## Materials and methods

*C. chinense* seeds (from dark red fruit) were obtained from a local field at Rüziephema village, Nagaland, India. The seeds were washed thoroughly in running tap water, then treated with 2% Labolene (v/v) for 10 min and finally rinsed five times with distilled water. These were then surface sterilized with 0.1% HgCl_2_ (Himedia) for 5 min followed by several rinses with sterile distilled water. The sterilized seeds were inoculated on MS medium (Murashige and Skoog 1962) containing 3% (w/v) sucrose and 0.8% (w/v) agar, pH was adjusted to 5.8. Six-week-old in vitro germinated seedlings were used as the source of explants. Shoot tips (1–2 cm) and nodal segments (1.0 cm) were excised from these seedlings and implanted in the culture medium. The shoot bud induction medium was supplemented with various concentrations of either 6-benzyl aminopurine, BAP (4.44–71.04 μM) or Thidiazuron, TDZ (2.27–36.32 μM). All media were autoclaved at 121 °C, 1.05 kg cm^−2^ pressure for 15 min. Cultures were incubated at room temperature of 25 ± 2 °C, 14/10-h photoperiod with an irradiance of 62.2 μmol m^−2^ s^−1^ provided by cool white fluorescent tubes. The bud-forming capacity (BFC) was calculated based on the average number of buds and percentage of response of the explants (Tandon et al. 2007) as follows:

The effect of BAP and TDZ on BFC was evaluated by regression analysis. The elongated shoots (1.5–2.5 cm) were then excised and transferred to root induction medium containing various concentrations of either indole-3-acetic acid, IAA (2.28–6.84 μM) or α-naphthalene acetic acid, NAA (0.53–4.29 μM). The number and length of the roots were recorded after 2 weeks of incubation. The well-rooted shoots were removed and washed thoroughly with distilled water. The plantlets were transplanted in perforated plastic cups filled with potting mixture of soil and cow dung (1:1) and irrigated with water daily in a green house. Finally, the hardened plants were transferred to earthen pots. All experiments were carried out with six replicates each and data were analyzed using one-way analysis of variance (ANOVA) at the 0.05 significance level in JMP^®^ version 7.0.1 (SAS Institute, Cary, NC, USA).

The significant differences among the means were assessed by Tukey’s Honestly Significant Difference (HSD) test used post-hoc on significant findings. Regression analysis for BFC index was performed using SPSS version 7.5.

## Results and discussion

Despite the economic importance of chili peppers, work on development of plant regeneration systems for *Capsicum* species has not progressed as in the case of several other solanaceous crops. In vitro regeneration of *Capsicum* species is reported to be difficult (Ochoa-alejo and Ramirez-malagon 2001).

**Fig. 1:**
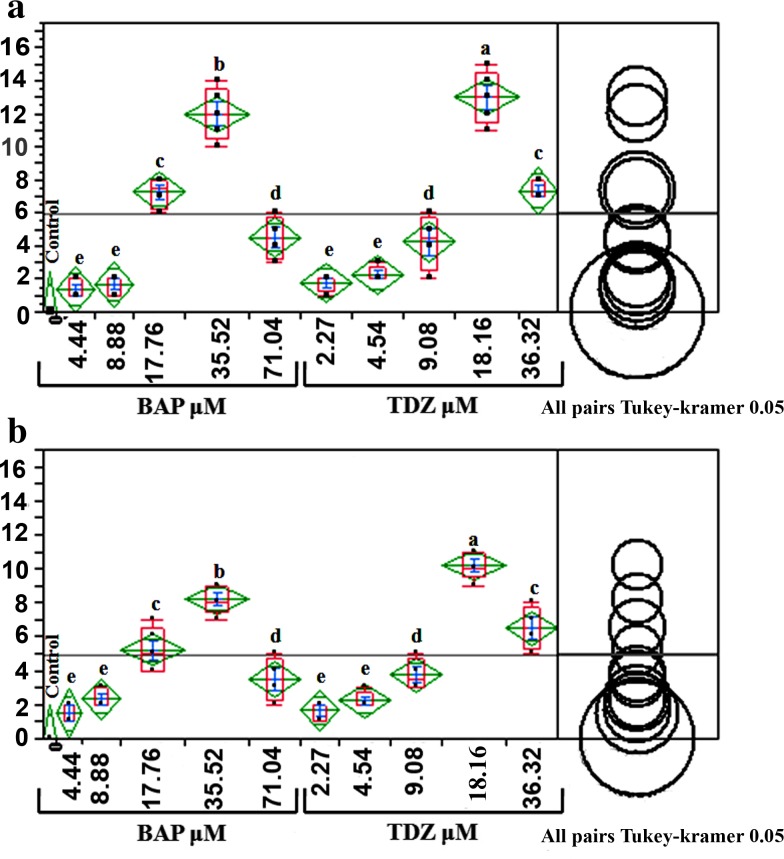
Effect of 6-benzylaminopurine and thidiazuron on shoot induction from nodal segments (**a**) and shoot tips (**b**) of *Capsicum chinense* Jacq. cv. Naga King Chili. in MS medium. Data scored after 5 weeks of culture. Tukey’s post-hoc test shows means that are not significantly different grouped by the same letter

**Fig. 2:**
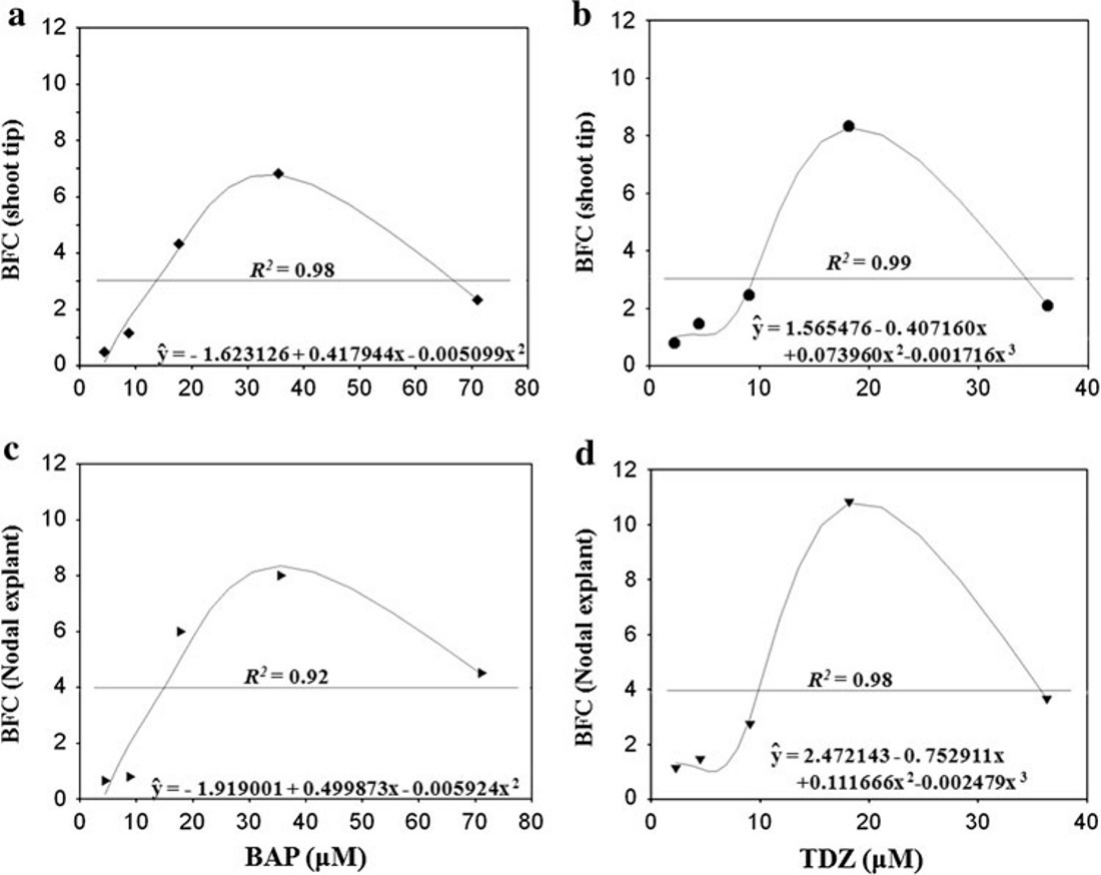
Effect of either BAP (4.44, 8.88, 17.76, 35.52 and 71.04 μM) or TDZ (2.27, 4.54, 9.08, 18.16 and 36.32 μM) concentration on bud-forming capacity from *C. chinense* shoot tip (**a**, **b**) and nodal explants (**c**, **d**), after 5 weeks of culture

**Fig. 3:**
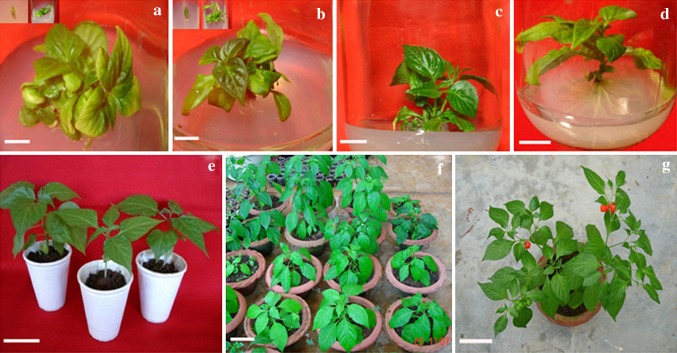
In vitro regeneration in *Capsicum chinense* Jacq. cv. Naga King Chili. **a** Shoot induction from nodal explants in MS +18.16 μM TDZ after 5 weeks (*Bar* 1 cm). **b** Shoot induction from shoot tip explants in MS +18.16 μM TDZ after 5 weeks (*Bar* 1 cm). **c** Elongated plantlets of regenerated shoots (*Bar* 1 cm). **d** Rooting of in vitro regenerated shoots after 3 weeks of culture (*Bar* 1 cm). **e** Regenerated hardened plantlets (*Bar* 3 cm). **f** Regenerated plantlet transferred to earthen pot (*Bar* 4 cm). **g** Regenerated plantlets bearing healthy fruit

In the present study, in vitro plantlet regeneration from shoot tips and nodal segments, cultured in MS medium supplemented with various concentrations of cytokinins (BAP or TDZ) alone are reported for *C. chinense*. TDZ is a phenyl urea that was originally developed as a cotton defoliant but has gained importance as a potent plant growth regulator for in vitro propagation systems of various crops (Fiola et al. 1990; Hutchinson et al. 1996). Based on review of available literature, very few reports exist on successful application of TDZ for rapid and efficient propagation of *C. annuum* (Venkataiah et al. 2003; Ahmad et al. 2006). Continuous exposure of explants to high concentration of TDZ is reported to be inhibitory/toxic, while low concentrations may not provide adequate stimulus (Huetterman and Preece 1993; Lu 1993; Hutchinson et al. 1996). In our study, the highest number of shoot buds (13 ± 0.70) with the BFC index of 10.8 was observed from nodal segments in MS medium containing 18.16 μM TDZ, followed by shoot tips (10 ± 0.37) with BFC index of 8.33 (Figs. 1a, b, 3a, b). The effectiveness of low dose (1.0 μM TDZ) on multiple shoot induction in *C. annuum* has been reported earlier (Ahmad et al. 2006); however, in the present investigation with *C. chinense*, the frequency of shoot formation was more favorable at higher doses of TDZ. This may be due to the recalcitrant nature of this species. Earlier workers reported the use of very high concentration of BAP for maximal shoot proliferation from shoot tip explants of capsicum (Christopher and Rajam 1994; Sanatombi and Sharma 2008). However, in the present investigation with 35.52 μM BAP alone, the maximum number of shoot induced from nodal segments was 12 ± 0.70 with BFC index of 7.99, followed by shoot tips (8.2 ± 0.37) with BFC index of 6.83. This increase in shoot formation is nearly twofold more than what has been reported earlier by Sanatombi and Sharma (2008). The BFC index is an efficient indicator of bud induction as it takes into consideration both the number of explants showing bud induction as well as the number of buds per explants (Tandon et al. 2007). An analysis of regression showed that regardless of the explants types used (nodal segment and shoot tip), the BFC gradually decreased at a concentration higher than 35.52 μM BAP. Whereas, in the case of TDZ as growth regulator, the most suitable TDZ level (18.16 μM TDZ) for *C. chinense* produced BFC index of 10.8 shoots per explant. Elevation of TDZ level beyond this concentration drastically reduced BFC index as indicated by regression analysis (Fig. 2a–d). This may be due to the toxic effect of TDZ at higher doses.

Unlike other solanaceous species, chili has been a recalcitrant species with regard to its capacity for in vitro plant regeneration (Liu et al. 1990). The rate of progress in *Capsicum* is relatively slower than other members of solanaceae because of its high genotypic dependence and recalcitrant nature. *Capsicum* is a recalcitrant plant in terms of in vitro cell, tissue and organ differentiation (Kothari et al. 2010). Direct organogenesis has been the most frequently used morphogenic route for in vitro chili plant regeneration. However, the major problem faced to achieve this goal has been the failure of elongation of the induced shoot buds (Ochoa-alejo and Ramirez-malagon 2001). Because of ill-defined buds, leafy or shoot like structures which fail to elongate, several attempts have been made by various workers to enhance elongation of shoot buds (Frank-Duchenne et al. 1998; Venkataiah et al. 2003; Sanatombi and Sharma 2008). In our study, maximum elongation of shoots (1.5–2.5 cm) was observed in MS Medium containing 18.16 μM TDZ or 35.52 μM BAP (Fig. 3c).

The elongated shoots were excised individually and cultured in MS medium containing either IAA (2.28–6.8 μM) or NAA (0.53–4.29 μM) for root induction. The regenerated shoots rooted within 2 weeks of treatment but with different rooting frequency in each treatment. The effects of auxins on root induction in in vitro regenerated chili plantlets have been reported (Agrawal et al. 1989; Christopher and Rajam 1996). In the present investigation, the chili plantlets were best rooted in a medium containing 5.70 μM IAA, with the highest number (28.4 ± 0.50) and maximum length (4.18 ± 0.26 cm) of roots (Table 1; Fig. 3d). Our observations are consistent with the earlier finding in which IAA was successfully employed for rooting in *C. chinense* (Sanatombi and Sharma 2008). Among the two auxins tested, IAA resulted in more effective root induction as compared to NAA in which the roots were short and thick. The rooted plantlets after 2–3 weeks were transferred to potting mixture of a 1:1 mix of soil and cow dung and kept in green house for hardening and acclimatization (Fig. 3e), where they showed 90% survival. The regenerated plants did not show any variation in the morphology and growth as compared to the parent plant. The hardened plantlets thus were transplanted and established in earthen pots and bore normal fruits (Fig. 3f, g).

**Table 1:** Effect of auxins on rooting of shoots from shoot tips and nodal segments of *C. chinense* in MS medium

Auxins	Concentration (μM)	Number of roots	Length of roots (cm)	Response (%)
Control	–	–	–	–
IAA	2.28	14.0 ± 0.70c	1.5 ± 0.22c	100
	3.42	17.4 ± 0.67b	1.6 ± 0.18c	100
	4.56	18.2 ± 1.06b	2.4 ± 0.24b	100
	5.70	28.4 ± 0.50a	4.1 ± 0.26a	100
	6.8	12.8 ± 0.86cd	3.7 ± 0.30a	100
NAA	0.53	10.0 ± 0.70de	0.6 ± 0.05d	100
	1.07	10.2 ± 0.86de	0.5 ± 0.12d	100
	2.14	14.0 ± 0.70c	0.6 ± 0.12d	100
	3.22	10.2 ± 0.58de	0.2 ± 0.03d	90
	4.29	8.2 ± 0.58e	0.1 ± 0.02d	80

Data scored after 2 weeks of culture. Means + SE. Means followed by the same letters are not significantly different according to Tukey’s HSD

## Conclusion

The present study demonstrates a simple and promising protocol for in vitro plantlet regeneration of *C. chinense* from nodal segments and shoots tips which is nearly twofold more than what has been reported earlier. The use of either BAP or TDZ favored the development of plant. However, the use of TDZ at a concentration higher than 18.16 μM TDZ appeared to be toxic. IAA may be preferred over NAA for root induction of this species. This protocol may be applied for conservation and large-scale propagation of individual genotypes of this species of chili.
